# Tumor Selective Cytotoxic Action of a Thiomorpholin Hydroxamate Inhibitor (TMI-1) in Breast Cancer

**DOI:** 10.1371/journal.pone.0043409

**Published:** 2012-09-18

**Authors:** Lynda Mezil, Carole Berruyer-Pouyet, Olivier Cabaud, Emmanuelle Josselin, Sébastien Combes, Jean-Michel Brunel, Patrice Viens, Yves Collette, Daniel Birnbaum, Marc Lopez

**Affiliations:** 1 Centre de Recherche en Cancérologie de Marseille (CRCM), Aix-Marseille Univ, Marseille, France; 2 Inserm-U1068 (laboratoire d'oncologie moléculaire), CNRS-UMR7258, Marseille, France; 3 Inserm-U1068 (laboratoire iSCB), CNRS-UMR7258, Marseille, France; 4 Institut Paoli-Calmettes, Marseille, France; 5 Centre d'Immunologie de Marseille-Luminy (CIML), Aix-Marseille Univ, Campus de Luminy, case 906, Marseille, France; Faculty of Pharmacy, Ain Shams University, Egypt

## Abstract

**Background:**

Targeted therapies, associated with standard chemotherapies, have improved breast cancer care. However, primary and acquired resistances are frequently observed and the development of new concepts is needed. High-throughput approaches to identify new active and safe molecules with or without an “*a priori*” are currently developed. Also, repositioning already-approved drugs in cancer therapy is of growing interest. The thiomorpholine hydroxamate compound TMI-1 has been previously designed to inhibit metalloproteinase activity for the treatment of rheumatoid arthritis. We present here the repositioning of TMI-1 drug in breast cancer.

**Methodology/Principal Findings:**

We tested the effect of TMI-1 on luminal, basal and ERBB2-overexpressing breast tumor cell lines and on MMTV-ERBB2/neu tumor evolution. We measured the effects on i) cell survival, ii) cell cycle, iii) extrinsic and intrinsic apoptotic pathways, iv) association with doxorubicin, docetaxel and lapatinib, v) cancer stem cells compartment. In contrast with conventional cytotoxic drugs, TMI-1 was highly selective for tumor cells and cancer stem cells at submicromolar range. All non-malignant cells tested were resistant even at high concentration. TMI-1 was active on triple negative (TN) and ERBB2-overexpressing breast tumor cell lines, and was also highly efficient on human and murine “primary” ERBB2-overexpressing cells. Treatment of transgenic MMTV-ERBB2/neu mice with 100 mg/kg/day TMI-1 alone induced tumor apoptosis, inhibiting mammary gland tumor occurrence and development. No adverse effects were noticed during the treatment. This compound had a strong synergistic effect in association with docetaxel, doxorubicin and lapatinib. We showed that TMI-1 mediates its selective effects by caspase-dependent apoptosis. TMI-1 was efficient in 34/40 tumor cell lines of various origins (ED50: 0.6 µM to 12.5 µM).

**Conclusions/Significance:**

This is the first demonstration of the tumor selective cytotoxic action of a thiomorpholin hydroxamate compound. TMI-1 is a novel repositionable drug not only for the treatment of adverse prognosis breast cancers but also for other neoplasms.

## Introduction

Breast cancer is a heterogeneous disease whose evolution is difficult to predict. Breast cancers can be subdivided in three groups upon biological features and associated therapies: tumors expressing estrogen (ER) and/or progesterone (PR) receptors, tumors overexpressing ERBB2, and triple negative (TN) tumors that lack ER, PR and express no or normal ERBB2 levels [1]. This classification led to the advent of “targeted therapy” for hormone receptor-positive and for ERBB2-overexpressing breast cancers, respectively. No routinely used targeted therapy for TN breast cancers exists. Gene expression profiling has identified major molecular subtypes – luminal, basal and ERBB2 - that are grossly but not completely overlapping with these groups [1]. TN and ERBB2 tumors are the most aggressive breast cancers.

New therapeutics expected to target molecular pathways involved in tumor expansion and progression are in development. These targets comprise tyrosine kinase receptors, signaling pathways molecules, angiogenic factors and inhibitors of DNA repair [2]. Even though recent data have demonstrated a marked efficiency of these new targeted therapies, it remains challenging to identify eligible patients for a given therapy. Moreover, acquired resistance are frequently noted in advanced disease due to loss of target or activation of downstream or alternative signaling pathways [3]. Thus, combinations of conventional chemotherapy and radiotherapy are still the standard of care for breast cancer. Anthracycline/taxane-based neoadjuvant chemotherapy is routinely used for the treatment of the different breast cancer subtypes. Not only these treatments have adverse effects in patients but do not prevent relapses, which are now attributed to resistance of cancer stem cells to the drugs [4].

Ideally, optimal chemotherapeutic drugs in development should have a marked inhibitory effect towards the largest panel of cancer cells and cancer stem cells, and reduced or no toxicity towards normal cells both *in vitro* and in preclinical models. High-throughput approaches to identify such active molecules with or without an “*a priori*” are currently developed in many laboratories [5]. Also, drug repositioning has been recently considered as a real alternative and attractive means to rapidly reach cancer clinical trials [6].

Metalloproteinases play a role in multiple steps of tumor progression such as angiogenesis, local invasion, intravasation, extravasation and formation of distant metastases [7]. Metalloproteinases belong to three families, including two large ones, matrix metalloproteinases (MMPs) and a disintegrin and metalloproteinase family (ADAM). ADAM-17 metalloproteinase inhibitors have been described as promising agents in the treatment of breast and lung cancers. ADAM-17 is involved in the shedding of EGFR (Epidermal Growth Factor receptor) ligands and ERBB2 and its targeting leads to decreased ERBB signaling [8], [9]. An ADAM-17 inhibitor is currently undergoing early clinical trials **(**Friedman et al., cancer research, meeting abstract). When testing different ADAM metalloproteinase inhibitors we identified one compound, named TMI-1, with unexpected properties. TMI-1 is a dual inhibitor of MMP and ADAM metalloproteinases [10]. We found that, in contrast to other ADAM inhibitors of the same family, TMI-1 killed breast tumor cell lines and was efficient in pre-clinical models. TMI-1 effect was mediated by cell cycle inhibition and induction of caspase-dependent apoptosis. TMI-1 is a valuable and promising repositionable drug for the treatment of breast cancer and probably for other types of cancer. This molecule defines a new class of chemical compound for the treatment of cancer.

## Materials and Methods

### Animals

FVB/N-Tg(MMTVneu)202Mul transgenic mice were purchased from the Jackson Laboratory (Bar Harbor, Maine 04609 USA). FVB/N and C57BL/6 mice were purchased from the Centre d'Elevage Roger Janvier (Le Genest-St-Isle, France). All mice were kept in a specific pathogen-free mouse facility and handled according to the rules of “Décret no. 87–848 du 19/10/1987, Paris.” All experiments were performed in agreement with the French Guidelines for animal handling and protocols described in this study and were reviewed and approved by the local ethics committee. Project: Comité Ethique-Provence #14 (Coordinated by J. Nunes, M. Aurrand-Lions and P. Gibier).

### Cell lines

Cells were cultivated according to laboratory recommendations. The human breast carcinoma cell line L226 cell line was isolated from mouse xenografted with tumor biopsy (Mezil et al., in preparation). The patient gave written informed consent for xenograft protocol and cell line derivation. The study was reviewed and approved by the Comité d'Orientation Stratégique (COS) de l'Institut Paoli-Calmettes (Institutional Review Board IPC, Centre de Lutte contre le Cancer - Project: 09-003 - Date of approval: February, 22^nd^, 2009.

### Isolation of a murine mammary tumor epithelial cell line (TgNeu27)

Mammary gland tumor was removed from euthanized MMTV-neu mouse. Tumor was dissociated in a trypsin-EDTA 0.25% buffer (Invitrogen) for 30 min at 37°C. Dissociated cells were filtered then centrifugated at 1200 rpm for 10 min. Cells were plated in dishes in DMEM-F12 (Invitrogen) supplemented with 10% FCS and incubated at 37°C in 5% CO2. Cells were subcultivated as they reached high confluency. Characterization was done on the basis of transgene expression/activation and tumorigenicity (data not shown).

### Reagents

TMI-1, TMI-2, TMI-005 were from the Wyeth company. Marimastat, batimastat were purchased from TOCRIS bioscience. Tanomastat was purchased from Agouron Pharmaceuticals. TAPI-1 was purchased from Peptide International. Docetaxel, doxorubicin and carbonyl cyanide *m*-chlorophenylhydrazone (CCCP) were from Sigma-Aldrich. Dihydroethidium was from Molecular Probes. Lapatinib was from Sequoia Research Products, United Kingdom. Z-VAD-FMK, Z-IETD-FMK, annexin V and 7-AAD (7-Aminoactinomycin D) were purchased from BD pharmingen. Anti-ERBB2 (sc-284) and anti-phosphotyrosine 1248 specific ERBB2 (PN2a) antibodies were from Santa-Cruz BT and Abcam companies, respectively.

### Synthesis of compound TMI-1-O-Me

In a 10 mL two necked round flask were placed at room temperature 10 mg of TMI-1 (2.51×10^−5^ mol) in 6 mL of anhydrous Methanol. 35 mg (2.51×10^−4^ mol) of K_2_CO_3_ were introduced followed by the addition of 36 mg (2.51×10^−4^ mol, 16 µL) of methyl iodide. Stirring was maintained at room temperature overnight. Water (20 mL) and ethyl acetate (20 mL) were added to allow phase separation and the aqueous phase was extracted twice. The bottom phase layer was dried over Na_2_SO_4_, filtered and concentrated in vacuo. After removal of the solvents, the crude residue was purified by chromatography on a silicagel column using CH_2_Cl_2_/Ethylacetate (1/1) eluent affording the expected product in 75% yield.

Compound TMI-1-O-Me: (3S)- N-Methoxy-4-{[4-[(2-butynyloxy)phenyl]sulfonyl}-2,2-dimethyl-3-thiomorpholine carboxamide. White solid; ^1^H NMR (DMSO *d*6): *δ*  = 8.98 (s, 1H), 8.13−8.11 (m, 2H), 7.31−7.28 (m,2H), 4.65 (s, 2H), 4.62−4.54 (m, 3H), 3.90−3.88 (m, 2H), 3.84 (s, 3H), 2.81−2.67 (m, 2H), 1.70 (m, 3H), 1.48 (s, 3H), 1.38 (s, 3H). ^13^C (DMSO *d*6): *δ* = 169.32, 162.09, 135.21, 128.08, 117.98, 81.82, 79.69, 66.23, 62.50, 54.75, 49.84, 27.36, 25.36, 22.51, 3.38. C_18_H_24_N_2_O_5_S_2_ m/z 412.524 (100%, (M+H^+^)).

### Characterization of cancer stem cells (CSC) population based on aldehyde dehydrogenase (ALDH) activity

To characterize CSCs, the Aldefluor kit (StemCell Technologies) was used. Briefly, cells were incubated in Aldefluor assay buffer containing ALDH substrate (BAAA (BODIPY® - aminoacetaldehyde), 1 µmol/L per 1×10^6^ cells). Negative control was done in the presence of diethylaminobenzaldehyde (DEAB), a specific ALDH inhibitor. Cells were then incubated for 40 min at 37°C. Cells were analyzed with the LSRII flow cytometer (Becton-Dickinson). PI (Propidium Iodide) exclusion was used to gate viable cells.

### Characterization of cancer stem cells population based on tumorsphere formation

To obtain primary tumorspheres, cells were plated in ultralow attachment plates (Corning) at a density of 1000 cells/mL. Cells were grown in a serum-free mammary epithelial growth medium (MEBM, Lonza), supplemented with B27 (GIBCO), 20 ng/mL EGF, 2 mg/mL hydrocortisone, 2 mg/mL insulin actrapid and 50 mM β-mercaptoethanol (GIBCO). To obtain secondary tumorspheres, primary tumorspheres were collected by centrifugation and dissociated enzymatically (10 min at 37°C in 0.05% trypsin, Invitrogen) and mechanically, using a fire-polished Pasteur pipette. Cells were counted and seeded in ultralow attachment plates (Corning) at a density of 1000 cells/mL.

### Cell growth/viability measurement

To analyze the effect of different drugs, cell growth was measured using the alamarBlue staining procedure as recommended by the manufacturer (Biosource). The test incorporates a fluorescent oxidation-reduction indicator. Fluorescence intensity is proportional to cellular metabolic reduction. Experiments were done by incubating 3000 cells/well in triplicate at Day 0 in 96 well plates. AlamarBlue was measured at Day 5 by incubating 1/10 volume of alamarBlue solution for 2 h at 37°C and read at 595 nm (FLUOstar Optima, BMG Labtech).

### Cell cycle analysis

SUM149 cells (10^6^) were incubated with TMI-1 for 48 h at indicated concentrations. Cells were treated according to the manufacturer's recommendations (BD Pharmingen, BrdU (Bromodeoxyuridin) Flow Kit). FITC conjugated BrdU antibody was incubated for 20 min at room temperature. Cells were washed and resuspended in a buffer containing 7-AAD. Cell cycle distribution was analyzed with a FACScalibur flow-cytometer (Becton-Dickinson). Analysis was done using the BD CellQuest flow cytometry analysis software.

### Analysis of caspase activity

Caspase activities were determined using commercially available kits. For the measurement of caspase-3/7 activity, 10^5^ cells were incubated in triplicate in 96 plates. TMI-1 at indicated concentrations was incubated for 24 h at 37°C. Activity was determined using luminescence Caspase-Glo Assays (Promega) according to the manufacturer's instruction. For the measurement of caspase-8 and caspase-9, 10^6^ cells/mL were incubated with TMI-1 at indicated concentrations for 48 h at 37°C. One µL of Red-IETD-FMK (caspase-8 test) or Red-LEHD-FMK (caspase-9 test) was added for 1 h at 37°C according to the manufacturer's recommendations (Biovision). Cells were washed then analyzed with the LSRII flow cytometer.

### Measurement of ROS production

Intracellular generation of ROS was measured using dihydroethidium. Cells (10^6^) were treated with TMI-1 or CCCP at indicated concentrations for 48 h. Cells were trypsinized then stained with 5 µM dihydroethidium for 30 min at 37°C. Ethidium fluorescence intensity resulting from dihydroethidium oxidation was measured using the LSRII flow cytometer.

### Western blot analysis

Cells and tumors were lyzed in ice-cold lysis buffer containing 50 mM Hepes, pH 7.5, 150 mM NaCl, 1.5 mM MgCl_2_, 1 mM EGTA, 1% Triton X-100, 0.2 mM sodium ortho*-*vanadate and 10% glycerol. “Complete” Protease Inhibitor Cocktail was added to cold lysis buffer as recommended by the manufacturer (Roche Diagnostics).

Cell lysates (25 µg) were separated using NuPAGE 4–12% Novex Bis-Tris gels according to manufacturer's recommendations (Invitrogen) and transferred onto Hybond™-C membrane (GE Healthcare Life Sciences). Membranes were blocked in PBS supplemented with 5% BSA for 1 h and then incubated with indicated antibodies. After washing, membranes were incubated with the secondary antibody conjugated with horseradish peroxidase (Pierce). The signal was detected using the ECL system (GE Healthcare Life Sciences).

### Transfection

Plasmids were transfected into SUM149 cells via the Nucleofector technology, as recommended by the manufacturer (Amaxa GmbH). Briefly, 5.10^6^ cells were resuspended in 100 µL Cell Line Nucleofector Solution V and nucleofected with 2 µg of each vector using program V-001 (Amaxa GmbH). Following nucleofection, the cells were immediately mixed with 500 µL of prewarmed F-12 Hams cell culture medium and transferred into 6-well plates containing 1.5 mL F-12 Hams medium per well. Cells were incubated at 37°C for 24 h before treatment. After 72 h of treatment, the cells were analyzed using a Beckman Dickinson LSR II flow cytometer.

### Mice treatment by TMI-1

MMTV-ERRB2/neu mice were treated intraperitoneally with TMI-1 (100 mg/kg/d) or with vehicle (0.9% NaCl, 0.5% methylcellulose, 2% Tween 80) for a period of 30 days. Injections started with the detection of the tumor. Tumor volume was calculated using the formula V = 0.52 (L×W^2^). Tumor volumes were combined when mice developed multiple tumors. Mice were weighted daily. After completion of the analysis, autopsy of mice was done.

### Measurement of apoptosis in mouse tumors

DNA fragmentation in apoptotic cells was analyzed by the TUNEL assay (ApopTag detection kit, (Millipore)) as recommended by the manufacturer. Briefly, 4 µM sections of paraffin-embedded fixed tissue were deparaffined with successive histolemon and ethanol washes, then treated with 20 µg/mL proteinase K for 15 min at RT. Endogenous peroxidase were quenched with 3% hydrogen peroxide. Digoxigenin-dNTPs were enzymatically added to the free 3′OH DNA termini by terminal deoxynucleotidyl transferase (TdT) and revealed by the peroxidase anti-digoxigenin antibody. Coloration was performed using the diaminobenzidine mixed substrate (Dako). Counterstaining was done with a solution of 1% methyl green for 5 min at RT. After distilled water and N-butanol washes, specimens were mounted in Pertex medium (CellPath). Observations were done using the Leica DMD108 digital microimaging device (Leica Microsystems GmbH).

### Drug combination analyses

Combination between TMI-1 and docetaxel, doxorubicin and lapatinib was analyzed by combining drugs at constant molar ratio. Concentration range for each drug was determined from effective dose (ED) 50 values calculated using nonlinear regression analysis. Cells were treated with serial dilutions of drug alone or in combination at constant molar ratio. Results were analyzed using the Chou and Talalay method that integrates ED50 and the shape of the dose-effect curve (Calcusyn software, Biosoft) [11]. The Combination Index (CI) was calculated to evaluate between additive (CI = 1) or synergism (CI<1) or antagonism (CI>1) resulting from drug combinations.

### Statistical analysis

Data are presented as mean ± s.e.m and were analysed using Mann-Whitney test or ANOVA, Bonferroni's Multiple Comparison test (Prism software). *P<0.05* was considered statistically significant.

## Results

### A metalloproteinase inhibitor inhibits the growth of breast tumor cells

**Figure 1:**
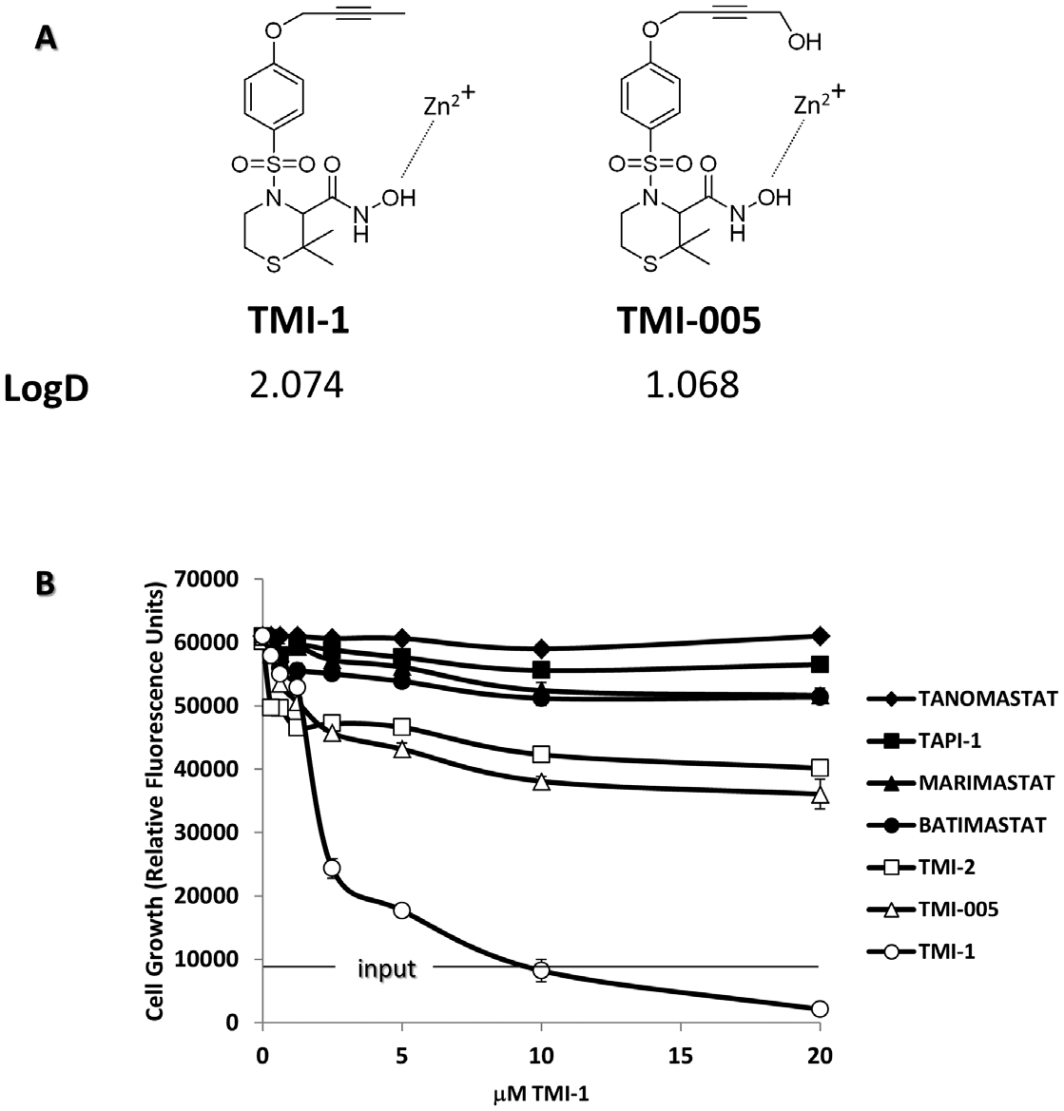
TMI-1 induces dose-dependent reduction of cell viability. **A:** Three different ADAM-17 inhibitors were used in the study. Chemical structures of TMI-1, TMI-005 and TMI-2 are represented. Interaction between hydroxamate moiety and enzymatic catalytic Zn-binding site is shown. LogD was calculated using the mean value obtained from three different prediction algorithms: ChemDraw, Mollinspi and AlogP. **B:** Cell viability was assessed on SUM149 cells at day 5. All compounds were diluted in DMSO at indicated concentrations. Cell viability assay was done with the Alamar blue staining kit. The “input” bar corresponds to the fluorescence level at Day 0. Experiments were done in triplicate for each concentration tested. Mean values +/− s.e.m is represented. These results are representative of three experiments.

Targeting ADAM-17 metalloproteinase activity has been recently described as a new alternative to treat breast tumors through inhibition of particular EGFR ligands and ERBB2 shedding. We tested a series of new ADAM-17 inhibitor compounds (TMI-1, TMI-2, TMI-005) for their potential ability to block tumor cell growth. These compounds belong to the class of sulphonamide hydroxamate agents. TMI-1 and TMI-005 have a closely related structure (Figure 1A). They are dual inhibitor for ADAM-17 and some other metalloproteinases and share similar inhibition spectra towards these metalloproteinases. TMI-005 (Apratastat) has been used in clinics for the treatment of a chronic inflammatory disease [12]. Concerning TMI-2, it is selective for ADAM-17 [13]. Other compounds previously used to treat patients with cancer were also tested. These are marimastat, tanomastat, and batimastat, which are commercially available matrix metalloproteinase (MMP) inhibitors [14]. In addition, TAPI-1, a broad spectrum MMP/ADAM dual inhibitor currently used *in vitro*, was tested.

We used the breast tumor cell line SUM149 (derived from an inflammatory breast carcinoma of basal subtype) to test the cell growth inhibition by these drugs at concentrations ranging from 0 µM to 20 µM. TMI-1 induced a marked dose-dependent inhibition of the growth of SUM149 cells with an ED50 value of 1.5 µM. At the end of the experiment, relative fluorescent level was below the input level suggesting a cytotoxic effect of TMI-1 on these cells (Figure 1B). TMI-2 and TMI-005, showed limited effect on SUM149 growth with no measurable ED50 value. The other compounds had no effect.

We extended TMI-1 inhibition tests to a panel of breast tumor cell lines representative of the different molecular subtypes (basal, ERBB2 and luminal). As shown in Table 1, TMI-1 inhibited 8 out of 9 tested tumor cell lines, with ED50 values comprised between 1.3 to 8.1 µM. These cells were faintly or not inhibited by TMI-2 or TMI-005 (data not shown). Interestingly, TMI-1 did not affect the viability of non tumoral and normal cells (n = 6). TMI-1 is thus a potent selective inhibitor for breast tumor cells of basal, ERBB2 and luminal molecular subtypes.

**Table 1:** TMI-1 inhibits cell viability and induces caspase-3/7 activity in different tumoral mammary cell lines.

Cells	Status	Cell growth	Caspase-3/-7
		inhibition	
		ED50 ( µM)	
BT-20	Basal	1.3	+
SUM149	Basal	1.5	+
MDA-MB-231	Basal	8.1	+
SK-BR-3	ERBB2	1.6	+
L226	ERBB2	2.0	+
SUM190	ERBB2	2.0	+
T47D	Luminal	2.5	+
Cama-1	Luminal	2.5	+
MCF-7	Luminal	>20	−
MCF10-A	Non tumoral	>20	−
HME-1	Normal Epithelium	>20	−
184A1	Normal Epithelium	>20	−
184B5	Normal Epithelium	>20	−
Endothelial	Primary Normal	>20	−
Fibroblast	Primary Normal	>20	−

**Figure 2:**
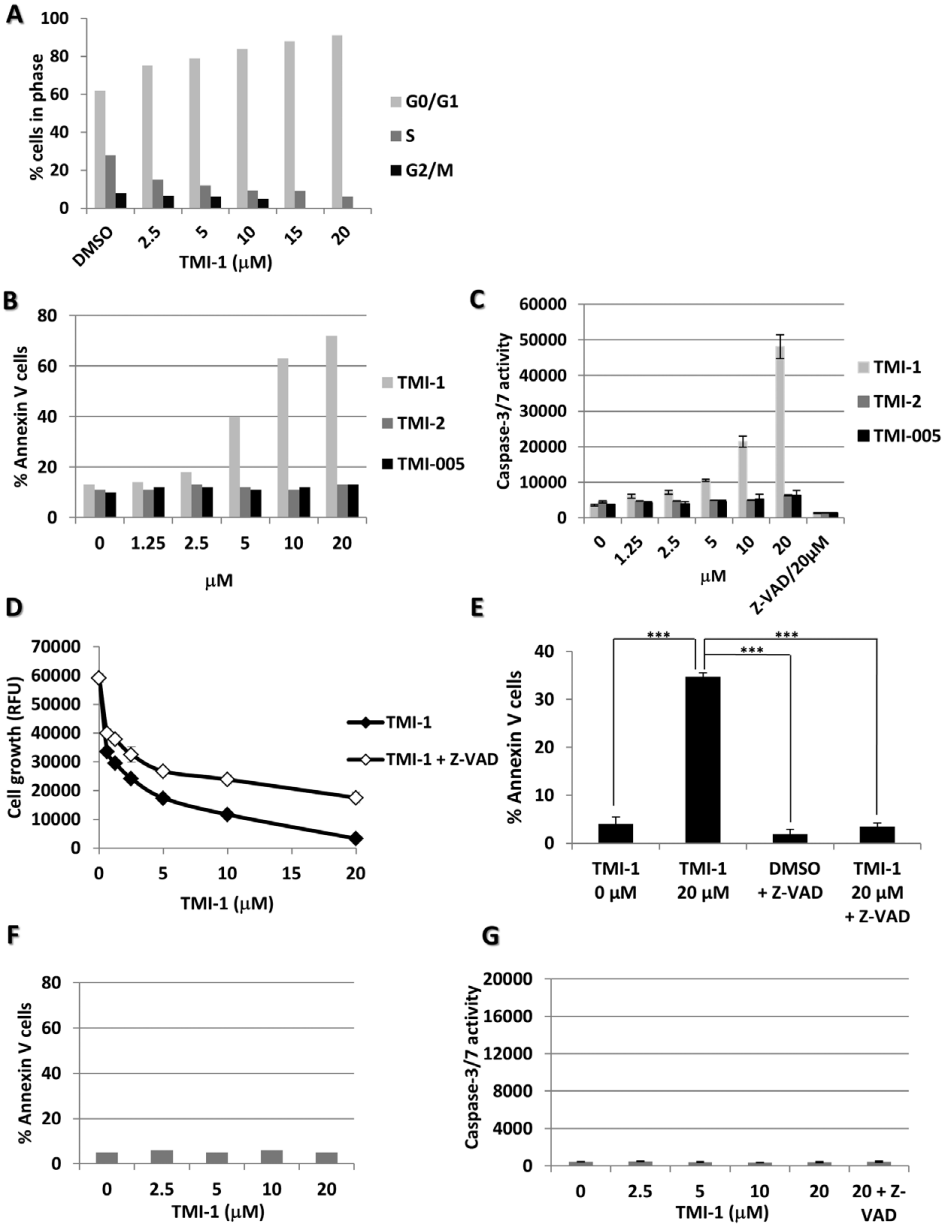
TMI-1 induces tumor cell death by apoptosis and cell cycle arrest. **A:** TMI-1 induces cell cycle arrest in the G0/G1 phase. SUM149 cells were treated for 48 h with TMI-1 or DMSO. Cells were stained, after BrdU incorporation, with anti BrdU-FITC antibody and 7-AAD and analyzed with the BD Facscalibur flow cytometer. **B:** TMI-1 induces dose-dependent apoptosis. Cell apoptosis was assessed by annexin V/7-AAD double staining after 48 h of treatment with 2.5 to 20 µM TMI-1, TMI-2 or TMI-005. Only TMI-1 induces apoptosis in SUM149 cells. **C:** TMI-1, not TMI-2 and TMI-005, induces a dose-dependent increase of caspase-3/7 activity after 24 h. General caspase inhibitor Z-VAD was included as positive control. **D:** TMI-1 induces caspase-dependent apoptosis. SUM149 cell viability was measured using the Alamar blue staining kit after 5 days of treatment with increasing doses of TMI-1, in the presence or absence of 20 µM Z-VAD. **E:** TMI-1 induces caspase-dependent apoptosis. Annexin V/7-AAD staining is inhibited in the presence of 20 µM Z-VAD. ANOVA, *P*<0.0005,*** Bonferroni's Multiple Comparison test. **F:** Apoptosis quantification in the non tumoral breast cell line MCF-10A treated by increasing doses of TMI-1. Treatment does not enhance annexin V/7-AAD staining. These results are reproduced four times. **G:** Caspase-3/7 activity in MCF-10A. Increasing doses of TMI-1 do not activate caspase-3/7 activity. Mean values +/− s.e.m. are represented in C, D, E, F. Experiments were done at least three times.

Breast tumoral and non tumoral epithelial cells as well as primary endothelial and fibroblastic cells were treated with TMI-1 (0.3125–20 µM) for 5 days. Cell viability assay was done with the Alamar blue staining kit. Caspase-3/7 activation was measured as in Figure 2. (+): Caspase-3/7 activity level increases (from 2 to 10 fold from control). (−): Caspase-3/7 activity level is invariant. Experiments were done in triplicate for each concentration tested. Molecular subtype status of each cell line and ED50 values are indicated. These results are representative of at least three independent experiments.

### TMI-1 treatment induces caspase-dependent apoptosis in human breast tumor cells

To characterize the impact of TMI-1 on cell viability we measured the consequence of treatment on cell cycle progression and apoptosis. TMI-1 treatment for 48 h resulted in a dose-dependent cell cycle arrest in the G0/G1 phase (Figure 2A and Figure S1), and a dose-dependent annexin V staining, with 5 µM TMI-1 inducing annexin V staining in 40% of treated cells following 48 h treatment (Figure 2B). No annexin V positive cells were detected following treatment with TMI-2 and TMI-005 (Figure 2B). Annexin V positive cells were detected 12 h after TMI-1 treatment, inferring that TMI-1 induced apoptosis is rather an early event (data not shown). Measurement of executioner caspase-3/7 activity showed a dose dependent activation by TMI-1 but not by TMI-2 and TMI-005 (Figure 2C). Treatment of cells by the non-selective caspase inhibitor Z-VAD-FMK markedly reduced TMI-1-induced cell growth inhibition (30%) and apoptosis (100%) (Figures 2D and 2E and Figure S2, left column). The residual growth inhibition observed in the presence of Z-VAD-FMK could correspond to TMI-1 inhibitory effect on cell cycle. Treatment of non-tumoral mammary epithelial cells MCF-10A did not induce annexin V staining or caspase-3/7 activity (Figures 2F and 2G). Actually, caspase-3/7 activity was not induced at all in all the non-tumoral and normal cell lines we tested (Table 1). In contrast, TMI-1 induced caspase-3/7 activity in all the sensitive breast tumor cell lines (Table 1).

It is of note that TMI-1-induced cell cycle inhibition, caspase activation and annexin V staining (measured at 48 h) were observable at micromolar range concentration compatible with TMI-1 concentration affecting cell viability (measured at 120 h). Associated with Z-VAD inhibition, these data suggest that cell cycle inhibition and apoptosis induction are early biological events triggered by TMI-1 treatment and responsible for tumor cell growth inhibition.

### TMI-1-induced apoptosis occurs through the extrinsic pathway

**Figure 3:**
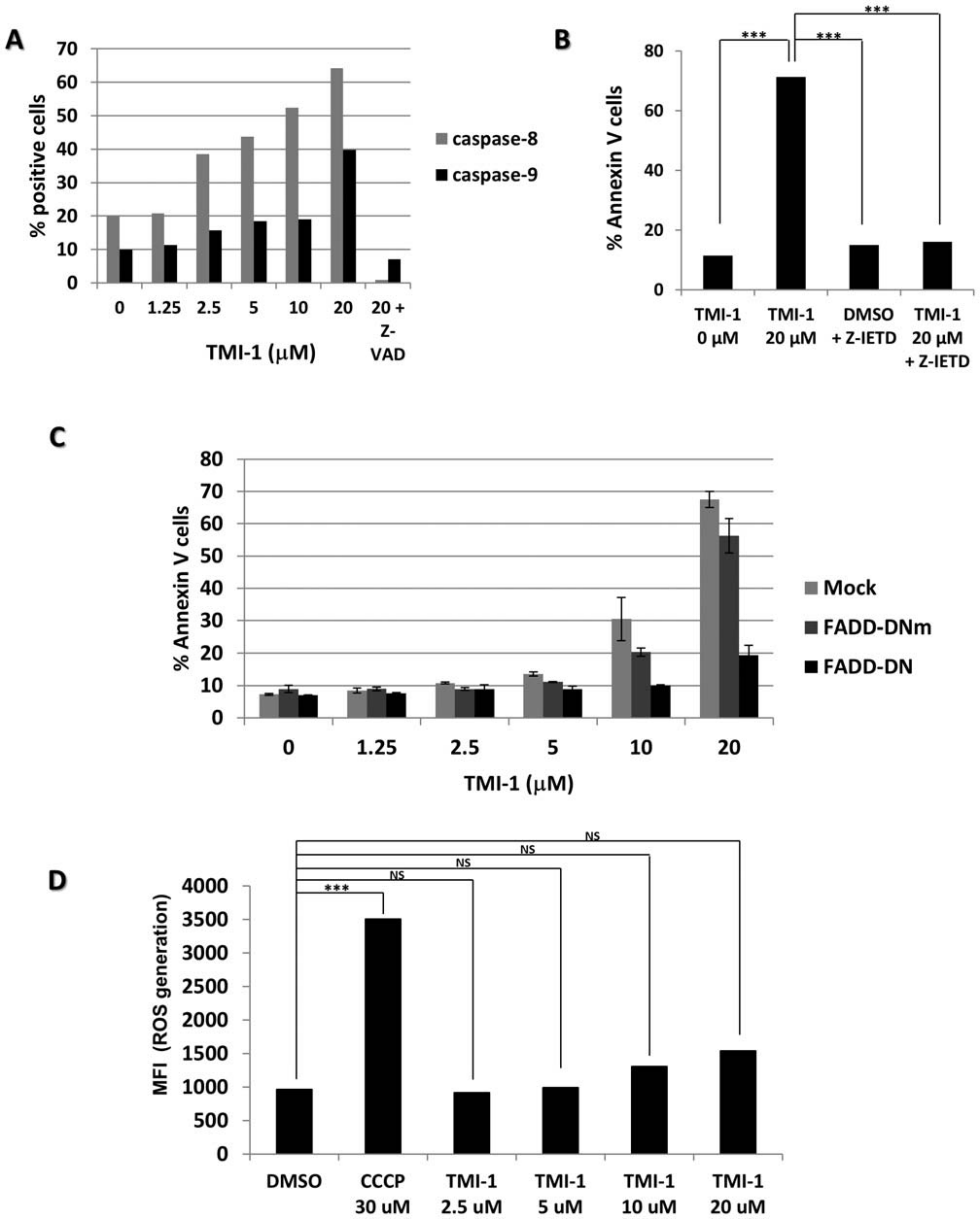
TMI-1-induced apoptosis requires mainly extrinsic death pathway. **A:** TMI-1 induces a dose–dependent activation of caspase-8 and caspase-9. SUM149 cells were treated for 48 h with 2.5 to 20 µM TMI-1. Treatment of SUM149 cells in the presence of Z-VAD (20 µM) was used as positive control. Caspase-8 and caspase-9 activities were assessed using pro-fluorescence LETD and LEHD tetrapeptide sequences, respectively. Results are presented as percent positive cells. **B:** TMI-1-induced apoptosis is caspase-8 dependent. SUM149 cells were treated with TMI-1 (20 µM), in the presence or absence of the specific caspase-8 inhibitor Z-IETD (50 µM). This experiment was measured by annexin V test and results are presented as percent annexin V positive cells. ANOVA, *P*<0.0005,*** Bonferroni's Multiple Comparison test. **C:** SUM149 cells expressing the FADD-DN construct protein are resistant to TMI-1-induced apoptosis. SUM149 cells were transfected with pB-FADD-DN wild type (FADD-DN) or pB-FADD-DN-muted (FADD-DNm) followed by treatment for 72 h with TMI-1 (1.25–20 µM). Apoptosis was determined by percentage of positive annexin V cell. Results shown are representative of 3 independent experiments; bars represent the mean +/− s.e.m.. Transfection efficiency determined by the use of pMax-GFP construct was >65%. **D:** TMI-1 induces slight intracellular accumulation of ROS. SUM149 cells were treated with TMI-1 (2.5–20 µM) for 48 h or CCCP (30 µM), used as positive control. Results were presented as mean of fluorescence intensity of hydroethidine oxydation. ANOVA, *P*<0.0005,*** Bonferroni's Multiple Comparison test.

Apoptosis induced by anticancer drugs involves the intrinsic mitochondrial pathway and/or elements of the death receptor signaling pathway, the so-called extrinsic pathway of apoptosis [15]. We sought to determine the mechanism by which TMI-1 triggers apoptosis in SUM149 cells. As shown in Figure 3A, we found a dose-dependent activation of caspase-8 and caspase-9 upon TMI-1 treatment.

Processed caspase-8 is associated with activation of the extrinsic pathway. The implication of the extrinsic pathway in TMI-1-induced cell death was verified using the Z-IETD caspase-8 inhibitor which blocks apoptosis at 95% (Figure 3B and Figure S2, bottom right). Consistent with these results, transfection of a dominant-negative form of FADD (FAS-Associated protein with Death Domain) (FADD-DN) in TMI-1-treated SUM149 cells induced an 82% reduction of annexin V staining. As expected, a FADD-DN mutant (FADD-DNm), which in contrast to FADD-DN did not compete with endogenous FADD binding, did not significantly reduced annexin V staining (Figure 3C). Together, these results demonstrate that TMI-1 induces apoptosis through the activation of the extrinsic pathway. This seems to be a general mechanism of action as we found activation of the extrinsic pathway in two other sensitive breast cell lines, BT20 and SK-BR-3 (Figure S3). However, this does not exclude a participation of the intrinsic pathway.

We tested the implication of the intrinsic pathway by measuring the enhancement of reactive oxygen species (ROS) generation. As shown in Figure 3D, we found a slight but reproducible dose-dependent increase of ROS generation in SUM149 cells. However, the uncoupler of oxidative phosphorylation CCCP clearly generated more ROS than TMI-1 (Figure 3D). We found no decrease in mitochondrial membrane potential using the diOC6 cationic lipophilic dye (data not shown). From these two results we can conclude that TMI-1 acts mainly throughout the extrinsic pathway.

### TMI-1 alone prevents MMTV-ErbB2/neu tumor evolution

**Figure 4:**
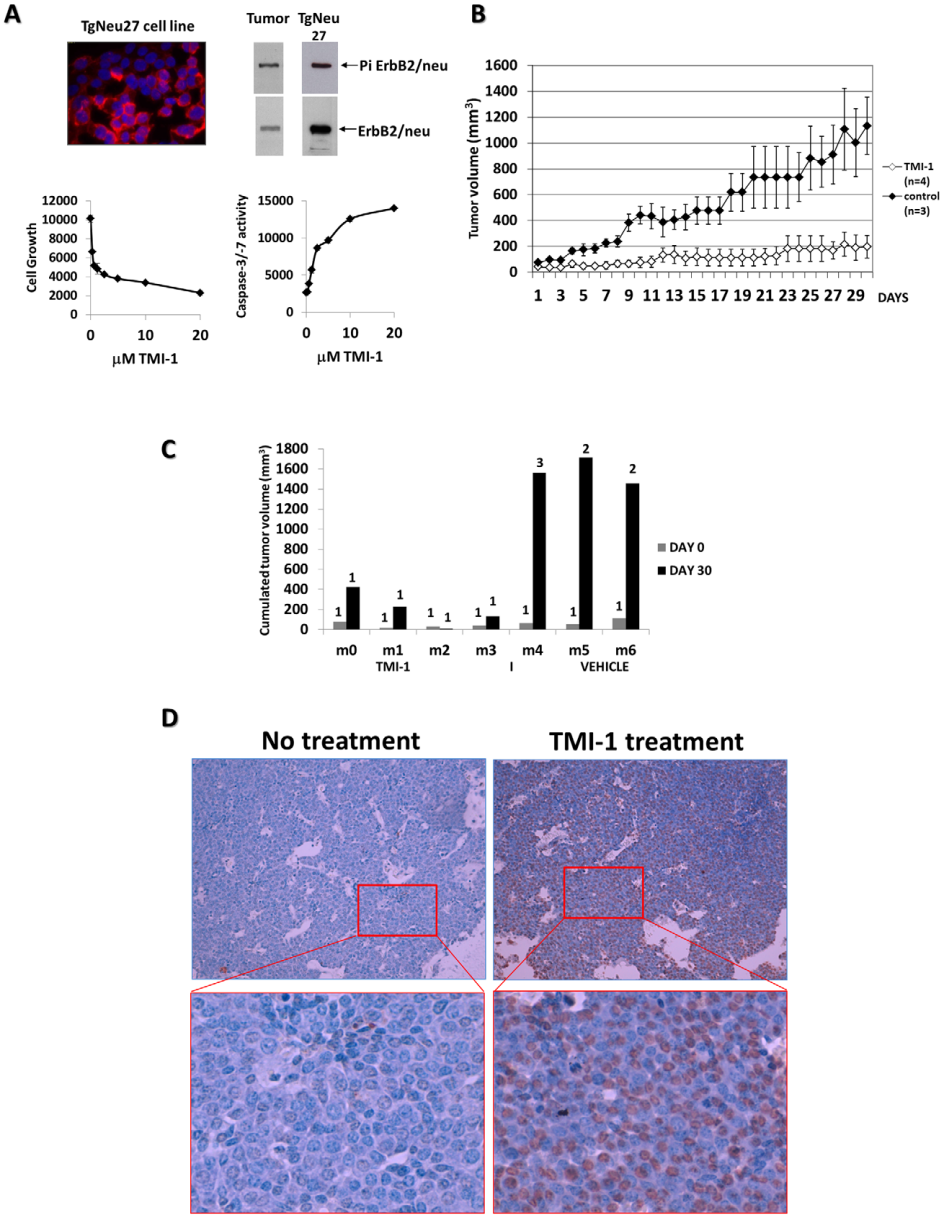
Anti-tumoral effect of TMI-1 in vivo. **A:** Effect of TMI-1 on the TgNeu27 cell line derived from a MMTV-ErbB2/neu tumor. ErbB2/neu expression by immunofluorescence (red staining) and western-blot analyzes showed expression and tyrosine phosphorylation of the transgene (top). This cell line is highly sensitive to TMI-1 as seen by cell growth inhibition and caspase-3/7 activation (bottom)**B:** MMTV-ErbB2/neu transgenic mice were injected daily IP with TMI-1 (n = 4) or with the vehicle of TMI-1 (n = 3). **C**: Tumor-cumulated volumes were assessed by adding the volumes of the primary tumors to the volumes of the other tumors developed in the same mouse. The number of tumors developed in the same mouse within the observation time (30 days) is indicated on the graph. **D:** TUNEL assay on tumors derived from MMTV-ErbB2/neu mice treated with vehicle (left) or TMI-1 (right) for 30 days (magnification: X100 (top), X400 (bottom)).

TMI-1 has been successfully used in mouse models of arthritis and kidney diseases to target and inhibit ADAM-17-mediated TNFα (Tumor Necrosis Factorα) and TGFα (Transforming Growth Factorα) release, respectively. TMI-1 pharmacokinetics in Balb/CJ mice indicated a C_max_ value of 3 µM for one dose of 50 mg/kg. This value is compatible with the ED50 we found *in vitro* (Table 1). This inhibitor is well tolerated in mice and rats at concentrations up to 200 mg/kg/day and 600 mg/kg/day, respectively. To test TMI-1 efficiency in a preclinical model of breast cancer, we focused on ERBB2-overexpressing breast cancer. Indeed, we found that ERBB2-overexpressing cell lines are sensitive to TMI-1 as was a cell line derived from MMTV-ErbB2/neu tumor overexpressing activated ErbB2/neu (TgNeu27) (Figure 4A and Table 1). To this end the transgenic MMTV-ErbB2/neu mouse was selected. This mouse model is a faithful model of spontaneous mammary gland carcinogenesis due to overexpression of the ErbB2/neu proto-oncogene and has been previously used to test the efficacy of drug therapies for breast cancer [16], [17], [18]. Because MMTV-ErbB2/neu mice progressively developed multifocal mammary tumors this model is also interesting to test tumor occurrence. Mice were treated at tumor onset for 30 days. Four mice (designated m0 to m3) were administered with TMI-1 at a dose of 100 mg/kg and three (m4 to m6) with vehicle alone. No adverse effects were noted during the treatment with the inhibitor as seen by mice behavior, body weight measurements and autopsy (data not shown). Daily administration of TMI-1 led to an 82% inhibition of mammary tumor growth compared to controls (Figure 4B). Interestingly, TMI-1 treatment prevented the occurrence of additional tumors (Figure 4C). Mice treated with the vehicle developed two or three tumors during the same period of observation, while no new tumors were detected in mice treated with TMI-1 (Figure 4C). In one case (mouse m2) we found a regression of the primary tumor upon TMI-1 treatment (Figure 4C). Apoptosis in tumors was measured in TMI-1 treated mice and compared with non-treated mice. Tumors from TMI-1 treated mice showed that approximately 60% of nuclei were positive by the TUNEL assay. No apoptosis was detected in tumors of mice treated with vehicle (Figure 4D). Together, these results showed that TMI-1 is efficient in ErbB2/neu mice model by inducing tumor apoptosis. Treatment of mice with TMI-1 slowed down mammary gland tumor growth and prevented tumor occurrence without detectable adverse effect.

### TMI-1 combines efficiently with and doxorubicin, docetaxel or lapatinib

**Figure 5:**
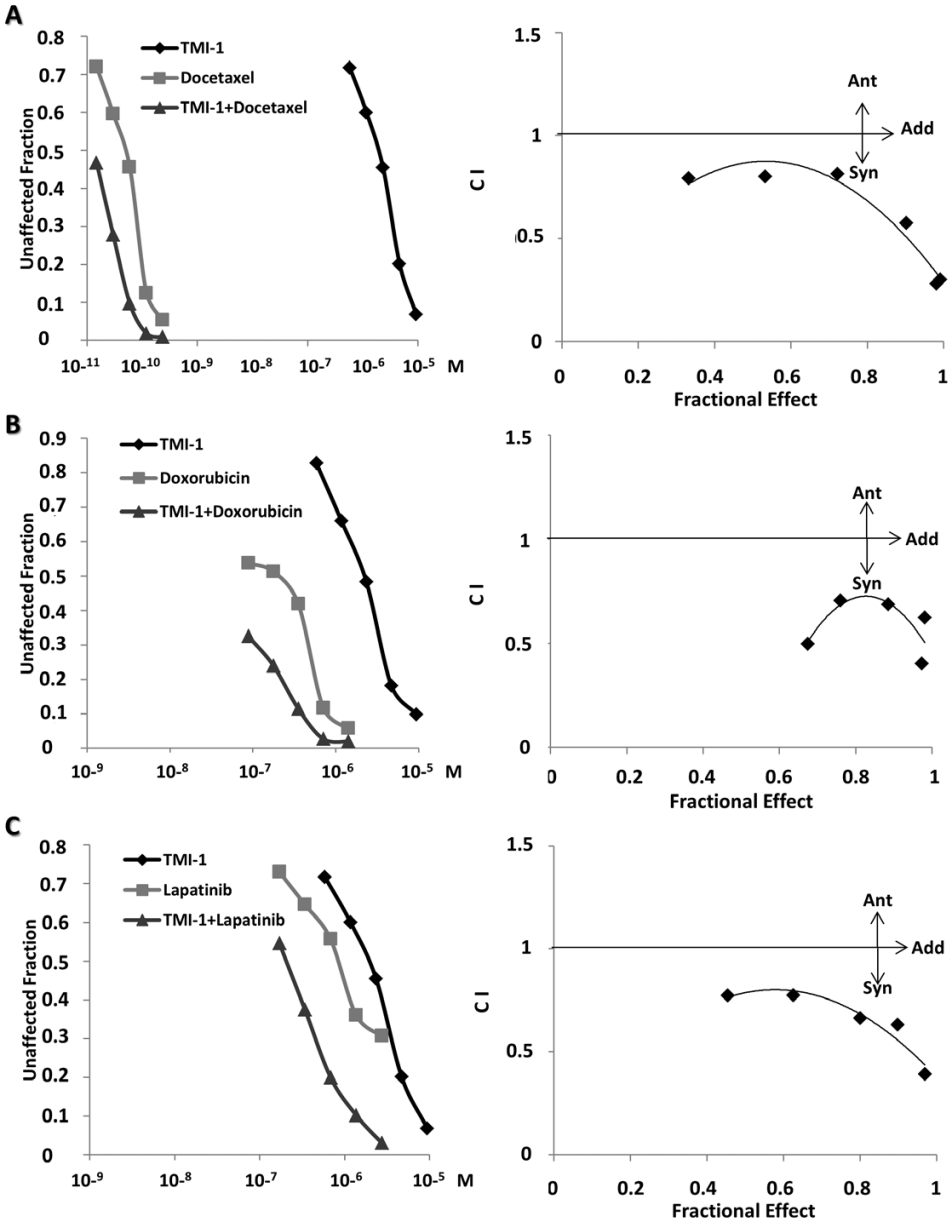
Association of TMI-1 with therapeutic drugs in vitro. SUM149 cells were incubated for 5 days with serial dilutions of TMI-1 (0.58–9.32 µM) and Docetaxel (14.87×10^−6^–238×10^−6^ µM) (**A**), doxorubicin (0.088–1.41 µM) (**B**), and lapatinib (0.17–2.72 µM) (**C**) individually or in combination at constant molar ratio. Determination between antagonism, additivity or synergism was evaluated using median-effect principle proposed by the Chou-Talalay method [11]. The CalcuSyn software was used to determine the CI.

Anthracyclines and taxanes are the standard of care for breast cancer treatment. They can be associated with each other or with other drugs, depending on histoclinical classification. The dual EGFR/ERBB2 tyrosine kinase inhibitor lapatinib is currently used as an adjuvant therapy in breast cancer. We sought to evaluate the interest of TMI-1 treatment in combination therapy with doxorubicin, docetaxel or lapatinib. The optimal molar ratio for each drug combination on the SUM149 cell line was determined based on their respective ED50 values. Cells were treated with serial dilutions of each drug either individually or in combination in a fixed ratio. The anti-tumoral action of TMI-1 was potentiated by docetaxel, doxorubicin and lapatinib (Figure 5A, B, C (left)), respectively.

The calculation of a combination index (CI) allows a quantitative definition for additive effect (CI = 1), synergism (CI<1), and antagonism (CI>1) in drug combinations. As shown in Figure 5A, B, C (right), synergism (CI<1) was observed for the three drug combinations and for the different ratio of concentrations. In some cases, CI values were low (<0.5) suggesting that, at these concentrations, the synergistic effect was marked. These results identify TMI-1 as a potent synergistic drug for breast cancer therapy. The data may indicate that mechanisms linked to TMI-1-induced cytotoxicity are different from the three other drugs tested.

### TMI-1 inhibits tumorsphere formation and Aldefluor-positive cells

Cancer stem cells (CSCs) are thought to contribute to tumor initiation, maintenance, resistance to therapy, and metastasis progression [19], [20]. Treatment with drugs enrich in resistant CSCs [4]. *In vitro*, breast CSCs form tumorspheres and express high level of aldehyde dehydrogenase-1 (ALDH1), which can be detected by using a commercial assay (CSCs are “Aldefluor” positive) [21], [22]. The SUM149 cell line has CSCs that show these properties and have been extensively used as *in vitro* and *in vivo* CSC model [23]. To be efficient on a breast tumor or cell line a therapeutic drug should target the CSCs. We thus wanted to determine whether TMI-1 was active on CSCs.

**Figure 6:**
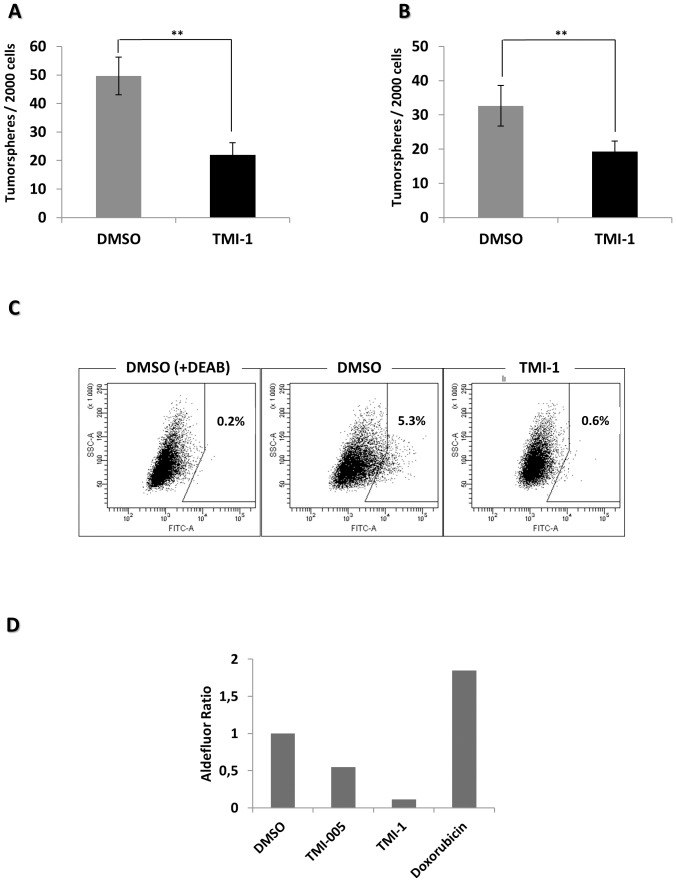
TMI-1 targeting of cancer stem cells. **A**: TMI-1 inhibits primary tumorspheres formation. SUM149 cells were treated with TMI-1 (2 µM) for 3 days. Cells were then cultured for 5 days to allow tumorsphere formation (see material and methods). Results are presented as the mean of tumorspheres counted. Bars represent the mean +/− s.e.m.. **P<0.005 as calculated using Mann-Whitney test. **B**: TMI-1 inhibits secondary tumorspheres formation. Dissociated primary tumorspheres were cultured for 5 days. Results are presented as the mean of tumorspheres counted. Bars represent the mean +/− s.e.m.. **P<0.005 as calculated using Mann-Whitney test. **C**: TMI-1 affects the pool of the ALDH-1 positive population. SUM149 cells were treated with TMI-1 (2 µM), TMI-005 (2 µM) or doxorubicin (0.352 µM) for 3 days. Cells were stained with Aldefluor kit and analyzed by flow cytometry. DEAB inhibitor was used to block aldehyde dehydrogenase activity. **D**: Overall results are represented as ratio between percent ALDH+ cells in treated conditions vs percent ALDH+ cells in DMSO. These results are representative of four experiments.

To generate tumorspheres, SUM149 cells (pre-treated or not by TMI-1) were plated in serum-free medium and low adherence culture conditions. TMI-1 pre-treatment reduced significantly the formation of tumorspheres in primary and secondary assays (Figures 6A and 6B). This impact of TMI-1 on CSCs was confirmed by the Aldefluor test that showed a marked decrease in Aldefluor-positive cells upon TMI-1 treatment (Figure 6C). In the same conditions, TMI-005 only slightly affected the pool of CSCs, whereas doxorubicin increased it (Figure 6D). Together, these data show that TMI-1 affects the pool of CSCs in SUM149 cells.

### TMI-1 cytotoxicity towards various tumor cell lines

Finally, to extend our results to several types of cancer, we tested the sensitivity of a total of 40 tumor cells from different origins. We integrated both ED50 value and the status of caspase-3/7 activity (+ or −) (Table 2). Based on ED50 and caspase-3/7 activity, TMI-1 was effective in 82% of the tested tumor cell lines. We defined that 44% of cell lines were highly sensitive to TMI-1 with ED50 values ranging from 0.6 µM to 2.5 µM. As seen previously for breast tumor cell lines (Table 1), caspase-3/7 were found active in all but one TMI-1 sensitive cell lines. The DU145 prostate cell line was the only cell line sensitive that showed absence of caspase-3/7 activity. Activation of caspase-3/7 by TMI-1 was found to strongly correlate with tumor cell growth inhibition. Reciprocally, no caspase-3/7 activity was detected in normal cells, which were all resistant to TMI-1 treatment.

**Table 2:** TMI-1 effects on cell viability and caspase-3/7 activation in 46 different cell types.

CELL LINE	TYPE	ERBB2	ED50	Casp-3/7
TOV-112D°	Ovary	+	0.6	+
A549°	NSCLC	−	0.8	+
PLCPRF5*	Hepatoma	−	1.1	+
ACHN*	Kidney	−	1.2	+
RL%	Lymphoma	ND	1.2	+
HUT78*	Lymphoblast	ND	1.3	+
BT-20°	Breast	−	1.3	+
SUM149∼	Breast	−	1.5	+
SK-BR-3°	Breast	+	1.6	+
A4573∞	Ewing Sarcoma	ND	1.8	+
TgNeu27¤	Breast	+	1.8	+
L226¤	Breast	+	2.0	+
SUM190∼	Breast	+	2.0	nd
U118*	Glioblastoma	ND	2.0	+
SW579*	Thyroid	ND	2.0	+
T47D°	Breast	−	2.5	+
Cama-1°	Breast	−	2.5	+
HGC27*	Stomach	ND	3.2	+
Karpas 299Ω	Lymphoma	ND	3.7	+
Messa%	Uterus	ND	3.9	+
PC-3∞	Prostate	−	4.5	+
DU145∞	Prostate	−	4.5	−
DLD-1#	Colon	−	4.5	+
MeWoδ	Melanoma	ND	5.0	+
U-2-OS'	Osteosarcoma	−	5.0	+
Calu-6*	Lung	−	5.0	+
HepG2*	Hepatoblast	−	5.4	+
Panc-1Ψ	Pancreas	−	7.0	+
A375δ	Melanoma	−	8.0	+
MDA-MB-231*	Breast	−	8.1	+
CLS354-4*	Head and Neck	ND	9.0	nd
AGS*	Stomach	−	10.0	+
H1299Ψ	NSCLC	−	12.5	+
OPM2∞	Myeloma	ND	17.0	−
HCT-116#	Colon	−	18.5	−
U87MG*	Glioblastoma	−	20.0	−
Hep2*	Larynx	−	20.0	−
MCF-7°	Breast	−	>20.0	−
BXPC3Ψ	Pancreas	−	>20.0	−
A498*	Kidney	−	>20.0	−
MCF10-A°	Epithelial	/	>20.0	−
HME-1°	Epithelial	/	>20.0	−
184A1°	Epithelial	/	>20.0	−
184B5°	Epithelial	/	>20.0	−
HUVEC+	Endothelial	/	>20.0	−
Fibroblast∧	Fibroblast	/	>20.0	−

Cell growth was assessed as Fig. 1B. ERBB2 expression: (+): high expression. (−): Low or no expression. (ND): No data available. For each cell line, ED50 value is indicated. Caspase-3/7 activation was measured as in Fig. 2. (+): Caspase-3/7 activity level increases. (−): Caspase-3/7 activity level is invariant. (nd): Not done. Cells were from:

° ATCC,

* CLS,

¤ CRCM,

# DSMZ,

+ Lonza. Gifts from:

∼ Dr S. P. Ethier (University of Michigan) [34],

∧ Dr C. Eaves (Terry Fox laboratory) [35], ‘ Arturo Londono-Vallejo (Institut Curie) [36],

% Lars P. Jordheim (Lyon I University) [37], [38],

Ω Bernard Payrastre (University of Medicine Toulouse-Purpan) [39],

Ψ Juan Iovanna (Aix-Marseille University) [40],

δ Sophie Tartare-Deckert (INSERM Unit 895, Nice) [41],

∞ Patrice Dubreuil (INSERM UMR1068, Marseille).

Thus, TMI-1 is a potent and selective inhibitor of tumor cell growth from different origins. No correlation with a specific type of tumor can be assessed from this analysis.

## Discussion

We present here a metalloproteinase inhibitor with unexpected properties in the field of anti-tumor therapy. TMI-1 works at sub-micromolar range concentration to induce cell cycle inhibition and tumor cell death via caspase-dependent apoptosis. We found that 82% of tumor cell lines tested are sensitive to TMI-1 compared to 0% for normal cells.

TMI-1 belongs to the class of thiomorpholine hydroxamate inhibitors. It was initially designed to treat patients with chronic inflammatory diseases through the inhibition of ADAM-17 metalloproteinase involved in the shedding of TNFα [10]. ADAM-17 is a cell surface protein that sheds multiple transmembrane proteins, especially EGFR ligands and ERBB2 [24]. ADAM-17 has thus been presented as a potent target in breast and lung cancers [25]. The ADAM-17 inhibitor INCB7839 has been shown to partially inhibit tumor cell growth at concentrations over 10 µM and synergized with targeted and cytotoxic drugs [26], [27]. Interestingly, this inhibitor is currently undergoing early clinical trials in association with Herceptin in ERBB2-positive advanced breast cancer patients.

Our data obtained with TMI-1 and its analogs TMI-005 and TMI-2 suggest that TMI-1 does not act through a mechanism strictly related to ADAM-17 inhibition. Indeed, we have established that these three inhibitors block the shedding of ADAM-17 targets such as TNFα, TGFα and amphiregulin (AREG) with the same efficacy (data not shown) whereas TMI-2 and TMI-005 only partially inhibit tumor cell growth and do not induce apoptosis. We also found that silencing expression of ADAM-17 results only in a moderate inhibition of cell growth, comparable to that observed with TMI-2 and TMI-005 (data not shown). We suggest from these data that apoptosis triggering by TMI-1 is not ADAM-17 dependent. This class of inhibitors acts by blocking the enzymatic catalytic Zn-binding site through their hydroxamate moiety. Interestingly, substitution of the hydroxamate moiety by a methyl group (TMI-1-O-CH3) (Figure S4A) profoundly altered the cytotoxic properties of TMI-1 (Figure S4B). This suggests that the thiomorpholine hydroxamate moiety could be implicated in TMI-1-induced apoptosis, through possible enzymatic inhibition. LogD values for the three inhibitors are different (TMI-1: 2.074, TMI-2: 0.012, TMI-005: 1.068). Thus, difference in lipophilic properties may also influence intracellular delivery of the drug through the plasma membrane, leading to differential targeting specificity and explain the marked difference observed between the closely related TMI-1 and TMI-005 to trigger apoptosis.

Of major interest is the fact that TMI-1 and TMI-005 have been found well tolerated in mice (up to 200 mg/kg/day/po) and rats (up to 600 mg/kg/day/po) [13], [28]. TMI-005 has been given to healthy volunteers up to 350 mg/po twice daily without any side effects [28]. The reason of this selectivity towards tumor cells is unclear. Recently, Moulick et al. described a small inhibitor that specifically targets tumor cells by blocking HSP90 only when engaged in cancer-specific but not in normal signaling networks [29]. Thus, there is increasing evidence that inhibitors may inhibit their target(s) according to the biological context. Tumor selectivity has been also described for HDAC (Histone deacetylase) inhibitor (HDACi) through TRAIL induction and triggering of the extrinsic apoptotic pathway [30]. HDACi belongs to a class of hydroxamate inhibitors that induce p21-dependent G1 cell cycle arrest. We found that TMI-1 did not induce p21, and did not act like an HDACi (data not shown). However, we have shown here that blocking the extrinsic pathway (FADD or caspase-8) led to apoptosis inhibition. This indicates that the apoptotic extrinsic pathway is activated upon TMI-1 treatment and that this pathway is necessary for triggering apoptosis.

Interestingly, TMI-1 inhibited 7 out of 9 breast tumor cell lines (ED50≤2.5 µM) irrespectively of their molecular subtypes. It is of note that all the ERRB2-overexpressing cell lines were sensitive: breast (SK-BR-3, SUM190) and ovarian cell lines (TOV-112D, see Table 2), the murine TgNeu27 “primary” cell line derived from a MMTV-ErbB2/neu tumor and the L226 “primary” cell line derived from a patient with inflammatory breast carcinoma. The L226 “primary” cell line overexpressed ERBB2 (Herceptest 3+) and recapitulated genomic, transcriptomic and phenotypic characteristics of the patient tumor (Mezil et al. in preparation). Interestingly, SUM190 and L226 were resistant to Herceptin *in vitro* ([31] and data not shown) meaning that TMI-1 may be an interesting alternative to treat the 70% of ERBB2 3+ patient that do not respond to Herceptin. Finally, TMI-1 slowed down tumor development and prevented the occurrence of new MMTV-ErbB2/neu mouse mammary tumors by inducing strong apoptosis. We noted tumor progression after completion of the treatment, strengthening the marked anti-tumoral *in vivo* effect of the drug used alone (data not shown). Together, these results suggest that patients with ERBB2-overexpressing tumors are eligible for TMI-1.

The inhibition of tumor occurrence observed in the mouse model is in accordance with the marked anti-CSCs effect of TMI-1. TMI-1 belongs to the (short) list of agents capable of targeting CSCs [20], [32]. Actually, some of these agents have been designed, like TMI-1, for the treatment of non-cancerous pathologies before drug repositioning towards cancer therapy (γ-secretase inhibitor (GSI) for Alzheimer's disease, metformin for diabetes type II, repertaxin in inflammation diseases) (Table S1). In the case of metformin, molecular target(s) is (are) not identified and the mechanism of action is not fully understood [33]. Unfortunately, metformin affects the growth of non-transformed cells. Several on-going randomized clinical trials that incorporate GSI and metformin as an adjuvant to chemotherapy are in progress.

## Conclusions

TMI-1 is a promising drug for the treatment of breast cancer and probably other neoplasms. TMI-1 is selective towards tumor cells and presents reduced toxicity *in vivo*. TMI-1 targets CSCs and differentiated tumor cells and is synergistic with chemo- and targeted-therapy agents.

TMI-1 could be used in different therapeutic protocols. For example, it could be administered in the adjuvant setting after removal of the primary tumor, to prevent tumor occurrence.

This work identifies a new class of chemical compound in the field of cancer therapeutics and raises the exciting possibility to find, define and refine new therapeutic target(s) and new chemical analogues.

## Supporting Information

Figure S1 TMI-1 induces a dose-dependent inhibition of cell cycle progression in G0/G1 phase. SUM149 cells were treated with the indicated doses of TMI-1 for 48 h. The different phases of cell cycle were defined by flow cytometry according to BrdU/7–AAD staining.
(TIF)

Figure S2 TMI-1 induces a dose-dependent apoptosis dependent of caspase activation. SUM149 cells were incubated with TMI-1 at the indicated concentration for 48 h. Apoptosis was measured by flow cytometry using annexin V/7-AAD staining.
(TIF)

Figure S3 TMI-1-induced apoptosis is caspase 8-dependent mechanism. SUM149, BT20 and SKBR3 cells were treated with TMI-1 (20 µM), specific caspase 8 inhibitor Z-IETD (50 µM) or both TMI-1 (20 µM) and Z-IETD (50 µM). This experiment was an annexin V test and results are presented as percent of annexin V positive cells. ANOVA, *P*<0.0005,*** Bonferroni's Multiple Comparison test.
(TIF)

Figure S4 Structure-Activity Relationship between TMI-1 and TMI-1-O-Me. TMI-1 hydroxamate group substitution by a methyl group leads to loss of cytotoxic activity. SUM149 cell growth was measured as presented in figure 1.
(TIF)

Table S1 Drug repositioning of molecules recently identified to target CSC.
(TIF)

## References

[1] Sorlie T, Perou CM, Tibshirani R, Aas T, Geisler S, et al (2001) Gene expression patterns of breast carcinomas distinguish tumor subclasses with clinical implications. Proc Natl Acad Sci U S A 98: 10869–10874. 11553815 10.1073/pnas.191367098 PMC58566

[2] Higgins MJ, Baselga J (2011) Targeted therapies for breast cancer. J Clin Invest 121: 3797–3803. 21965336 10.1172/JCI57152 PMC3195649

[3] Wong ST, Goodin S (2009) Overcoming drug resistance in patients with metastatic breast cancer. Pharmacotherapy 29: 954–965. 19637949 10.1592/phco.29.8.954

[4] Dave B, Chang J (2009) Treatment resistance in stem cells and breast cancer. J Mammary Gland Biol Neoplasia 14: 79–82. 19259795 10.1007/s10911-009-9117-9

[5] McDermott SP, Wicha MS (2010) Targeting breast cancer stem cells. Mol Oncol 4: 404–419. 20599450 10.1016/j.molonc.2010.06.005 PMC3023958

[6] Weir SJ, DeGennaro LJ, Austin CP (2012) Repurposing approved and abandoned drugs for the treatment and prevention of cancer through public-private partnership. Cancer Res 72: 1055–1058. 22246671 10.1158/0008-5472.CAN-11-3439 PMC3341848

[7] Overall CM, Lopez-Otin C (2002) Strategies for MMP inhibition in cancer: innovations for the post-trial era. Nat Rev Cancer 2: 657–672. 12209155 10.1038/nrc884

[8] Kenny PA, Bissell MJ (2007) Targeting TACE-dependent EGFR ligand shedding in breast cancer. J Clin Invest 117: 337–345. 17218988 10.1172/JCI29518 PMC1764856

[9] Zhou BB, Peyton M, He B, Liu C, Girard L, et al (2006) Targeting ADAM-mediated ligand cleavage to inhibit HER3 and EGFR pathways in non-small cell lung cancer. Cancer Cell 10: 39–50. 16843264 10.1016/j.ccr.2006.05.024 PMC4451119

[10] Zhang Y, Xu J, Levin J, Hegen M, Li G, et al (2004) Identification and characterization of 4-[[4-(2-butynyloxy)phenyl]sulfonyl]-N-hydroxy-2,2-dimethyl-(3S)thiomorpho linecarboxamide (TMI-1), a novel dual tumor necrosis factor-alpha-converting enzyme/matrix metalloprotease inhibitor for the treatment of rheumatoid arthritis. J Pharmacol Exp Ther 309: 348–355. 14718605 10.1124/jpet.103.059675

[11] Chou TC, Talalay P (1984) Quantitative analysis of dose-effect relationships: the combined effects of multiple drugs or enzyme inhibitors. Adv Enzyme Regul 22: 27–55. 6382953 10.1016/0065-2571(84)90007-4

[12] Moss ML, Sklair-Tavron L, Nudelman R (2008) Drug insight: tumor necrosis factor-converting enzyme as a pharmaceutical target for rheumatoid arthritis. Nat Clin Pract Rheumatol 4: 300–309. 18414459 10.1038/ncprheum0797

[13] Zhang Y, Hegen M, Xu J, Keith JC Jr, Jin G, et al (2004) Characterization of (2R, 3S)-2-([[4-(2-butynyloxy)phenyl]sulfonyl]amino)-N,3-dihydroxybutanamide, a potent and selective inhibitor of TNF-alpha converting enzyme. Int Immunopharmacol 4: 1845–1857. 15531300 10.1016/j.intimp.2004.08.003

[14] Coussens LM, Fingleton B, Matrisian LM (2002) Matrix metalloproteinase inhibitors and cancer: trials and tribulations. Science 295: 2387–2392. 11923519 10.1126/science.1067100

[15] Debatin KM, Krammer PH (2004) Death receptors in chemotherapy and cancer. Oncogene 23: 2950–2966. 15077156 10.1038/sj.onc.1207558

[16] Bouchard L, Lamarre L, Tremblay PJ, Jolicoeur P (1989) Stochastic appearance of mammary tumors in transgenic mice carrying the MMTV/c-neu oncogene. Cell 57: 931–936. 2567634 10.1016/0092-8674(89)90331-0

[17] Howe LR, Subbaramaiah K, Patel J, Masferrer JL, Deora A, et al (2002) Celecoxib, a selective cyclooxygenase 2 inhibitor, protects against human epidermal growth factor receptor 2 (HER-2)/neu-induced breast cancer. Cancer Res 62: 5405–5407. 12359744

[18] Liu M, Howes A, Lesperance J, Stallcup WB, Hauser CA, et al (2005) Antitumor activity of rapamycin in a transgenic mouse model of ErbB2-dependent human breast cancer. Cancer Res 65: 5325–5336. 15958580 10.1158/0008-5472.CAN-04-4589

[19] Charafe-Jauffret E, Ginestier C, Iovino F, Tarpin C, Diebel M, et al (2010) Aldehyde dehydrogenase 1-positive cancer stem cells mediate metastasis and poor clinical outcome in inflammatory breast cancer. Clin Cancer Res 16: 45–55. 20028757 10.1158/1078-0432.CCR-09-1630 PMC2874875

[20] Liu S, Wicha MS (2010) Targeting breast cancer stem cells. J Clin Oncol 28: 4006–4012. 20498387 10.1200/JCO.2009.27.5388 PMC4872314

[21] Dontu G, Abdallah WM, Foley JM, Jackson KW, Clarke MF, et al (2003) In vitro propagation and transcriptional profiling of human mammary stem/progenitor cells. Genes Dev 17: 1253–1270. 12756227 10.1101/gad.1061803 PMC196056

[22] Ginestier C, Hur MH, Charafe-Jauffret E, Monville F, Dutcher J, et al (2007) ALDH1 is a marker of normal and malignant human mammary stem cells and a predictor of poor clinical outcome. Cell Stem Cell 1: 555–567. 18371393 10.1016/j.stem.2007.08.014 PMC2423808

[23] Charafe-Jauffret E, Ginestier C, Iovino F, Wicinski J, Cervera N, et al (2009) Breast cancer cell lines contain functional cancer stem cells with metastatic capacity and a distinct molecular signature. Cancer Res 69: 1302–1313. 19190339 10.1158/0008-5472.CAN-08-2741 PMC2819227

[24] Reiss K, Saftig P (2009) The “a disintegrin and metalloprotease” (ADAM) family of sheddases: physiological and cellular functions. Semin Cell Dev Biol 20: 126–137. 19049889 10.1016/j.semcdb.2008.11.002

[25] Kenny PA (2007) Tackling EGFR signaling with TACE antagonists: a rational target for metalloprotease inhibitors in cancer. Expert Opin Ther Targets 11: 1287–1298. 17907959 10.1517/14728222.11.10.1287

[26] Fridman JS, Caulder E, Hansbury M, Liu X, Yang G, et al (2007) Selective inhibition of ADAM metalloproteases as a novel approach for modulating ErbB pathways in cancer. Clin Cancer Res 13: 1892–1902. 17363546 10.1158/1078-0432.CCR-06-2116

[27] Witters L, Scherle P, Friedman S, Fridman J, Caulder E, et al (2008) Synergistic inhibition with a dual epidermal growth factor receptor/HER-2/neu tyrosine kinase inhibitor and a disintegrin and metalloprotease inhibitor. Cancer Res 68: 7083–7089. 18757423 10.1158/0008-5472.CAN-08-0739

[28] Thabet MM, Huizinga TW (2006) Drug evaluation: apratastat, a novel TACE/MMP inhibitor for rheumatoid arthritis. Curr Opin Investig Drugs 7: 1014–1019. 17117591

[29] Moulick K, Ahn JH, Zong H, Rodina A, Cerchietti L, et al (2011) Affinity-based proteomics reveal cancer-specific networks coordinated by Hsp90. Nat Chem Biol 7: 818–826. 21946277 10.1038/nchembio.670 PMC3265389

[30] Nebbioso A, Clarke N, Voltz E, Germain E, Ambrosino C, et al (2005) Tumor-selective action of HDAC inhibitors involves TRAIL induction in acute myeloid leukemia cells. Nat Med 11: 77–84. 15619633 10.1038/nm1161

[31] Ginestier C, Adelaide J, Goncalves A, Repellini L, Sircoulomb F, et al (2007) ERBB2 phosphorylation and trastuzumab sensitivity of breast cancer cell lines. Oncogene 26: 7163–7169. 17525746 10.1038/sj.onc.1210528

[32] Hirsch HA, Iliopoulos D, Tsichlis PN, Struhl K (2009) Metformin selectively targets cancer stem cells, and acts together with chemotherapy to block tumor growth and prolong remission. Cancer Res 69: 7507–7511. 19752085 10.1158/0008-5472.CAN-09-2994 PMC2756324

[33] Bost F, Sahra IB, Le Marchand-Brustel Y, Tanti JF (2010) Metformin and cancer therapy. Curr Opin Oncol 24: 103–108. 10.1097/CCO.0b013e32834d8155 22123231

[34] Ignatoski KM, Ethier SP (1999) Constitutive activation of pp125fak in newly isolated human breast cancer cell lines. Breast Cancer Res Treat 54: 173–182. 10424408 10.1023/a:1006135331912

[35] Kuperwasser C, Chavarria T, Wu M, Magrane G, Gray JW, et al (2004) Reconstruction of functionally normal and malignant human breast tissues in mice. Proc Natl Acad Sci U S A 101: 4966–4971. 15051869 10.1073/pnas.0401064101 PMC387357

[36] Temime-Smaali N, Guittat L, Wenner T, Bayart E, Douarre C, et al (2008) Topoisomerase IIIalpha is required for normal proliferation and telomere stability in alternative lengthening of telomeres. EMBO J 27: 1513–1524. 18418389 10.1038/emboj.2008.74 PMC2396391

[37] Galmarini CM, Clarke ML, Jordheim L, Santos CL, Cros E, et al (2004) Resistance to gemcitabine in a human follicular lymphoma cell line is due to partial deletion of the deoxycytidine kinase gene. BMC Pharmacol 4: 8. 15157282 10.1186/1471-2210-4-8 PMC428575

[38] Jordheim LP, Galmarini CM, Dumontet C (2006) Gemcitabine resistance due to deoxycytidine kinase deficiency can be reverted by fruitfly deoxynucleoside kinase, DmdNK, in human uterine sarcoma cells. Cancer Chemother Pharmacol 58: 547–554. 16463058 10.1007/s00280-006-0195-8

[39] Cussac D, Greenland C, Roche S, Bai RY, Duyster J, et al (2004) Nucleophosmin-anaplastic lymphoma kinase of anaplastic large-cell lymphoma recruits, activates, and uses pp60c-src to mediate its mitogenicity. Blood 103: 1464–1471. 14563642 10.1182/blood-2003-04-1038

[40] Malicet C, Lesavre N, Vasseur S, Iovanna JL (2003) p8 inhibits the growth of human pancreatic cancer cells and its expression is induced through pathways involved in growth inhibition and repressed by factors promoting cell growth. Mol Cancer 2: 37. 14613582 10.1186/1476-4598-2-37 PMC280693

[41] Robert G, Gaggioli C, Bailet O, Chavey C, Abbe P, et al (2006) SPARC represses E-cadherin and induces mesenchymal transition during melanoma development. Cancer Res 66: 7516–7523. 16885349 10.1158/0008-5472.CAN-05-3189

