# Supplementary figures and images for: Tumor Selective Cytotoxic Action of a Thiomorpholin Hydroxamate Inhibitor (TMI-1) in Breast Cancer
Source: PLoS One. 2012 Sep 18;7(9):e43409. doi: 10.1371/journal.pone.0043409 (PMC3445597; doi:10.1371/journal.pone.0043409)

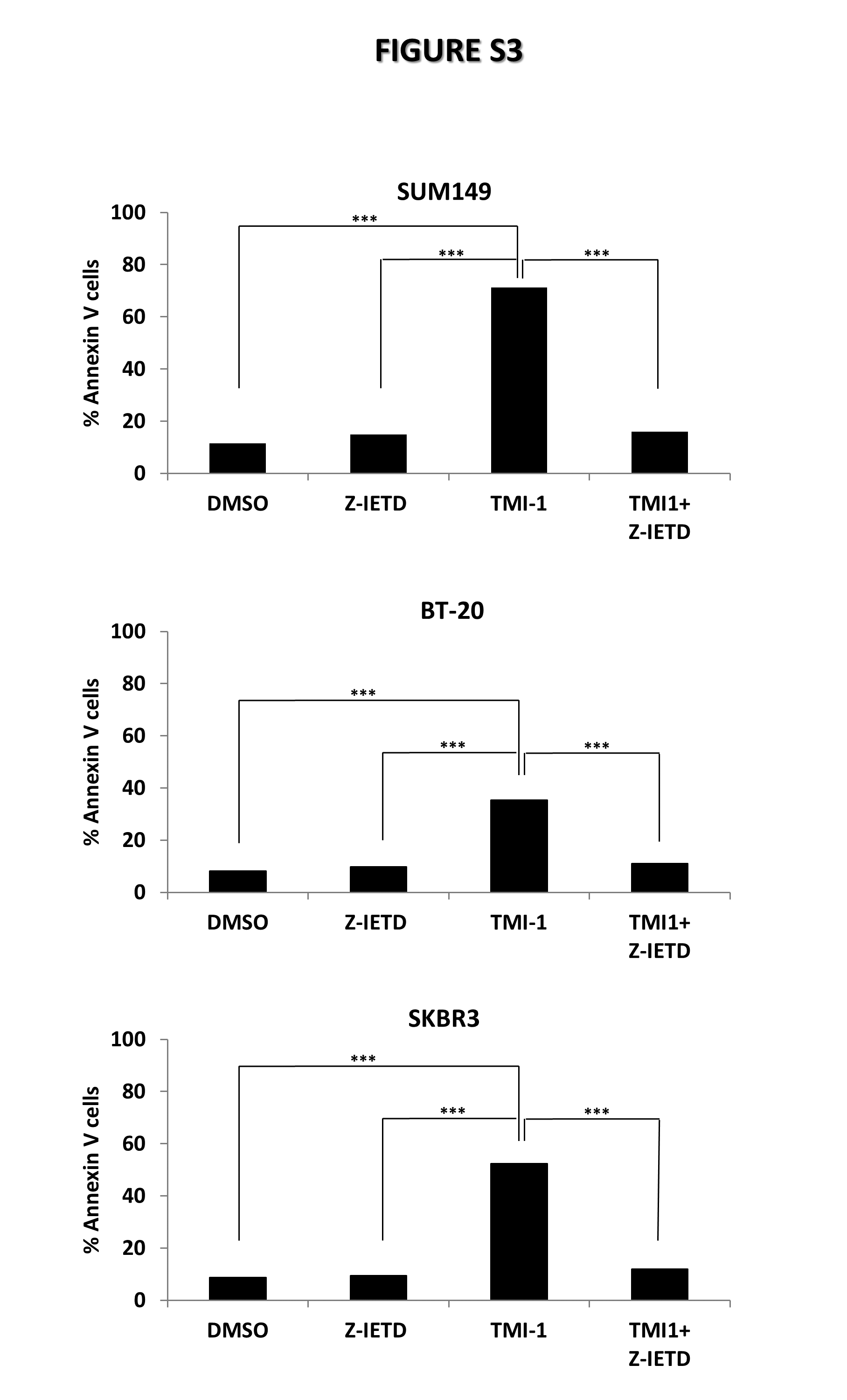

Supplement: Figure S3 — TMI-1-induced apoptosis is caspase 8-dependent mechanism. SUM149, BT20 and SKBR3 cells were treated with TMI-1 (20 µM), specific caspase 8 inhibitor Z-IETD (50 µM) or both TMI-1 (20 µM) and Z-IETD (50 µM). This experiment was an annexin V test and results are presented as percent of annexin V positive cells. ANOVA, P<0.0005,*** Bonferroni's Multiple Comparison test. (TIF) [file pone.0043409.s003.tif]

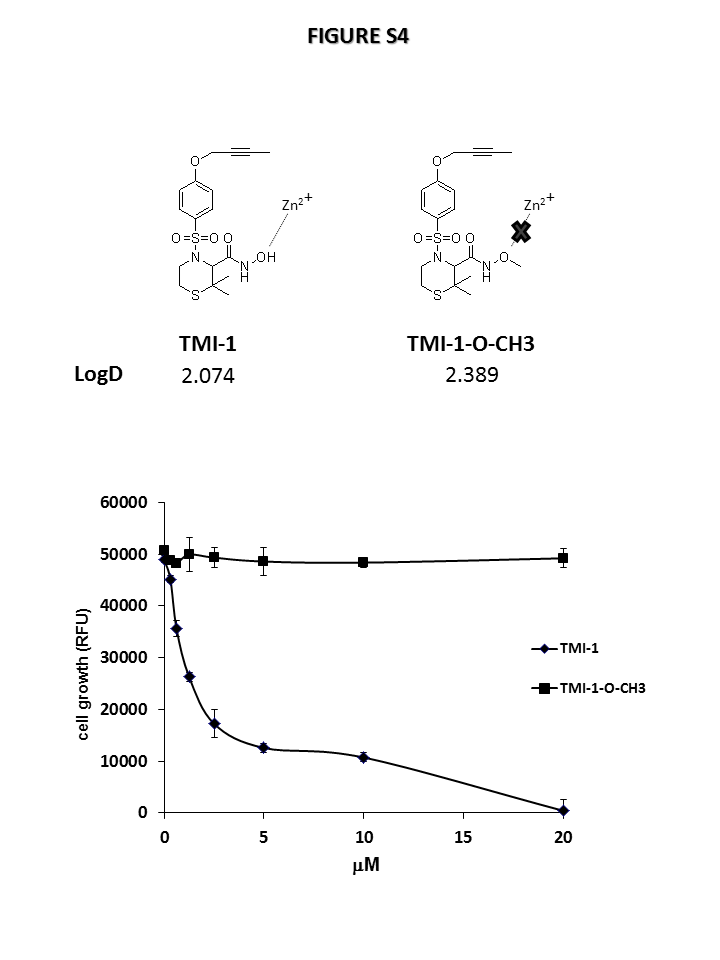

Supplement: Figure S4 — Structure-Activity Relationship between TMI-1 and TMI-1-O-Me. TMI-1 hydroxamate group substitution by a methyl group leads to loss of cytotoxic activity. SUM149 cell growth was measured as presented in figure 1. (TIF) [file pone.0043409.s004.tif]
